# *RARS1*‐related developmental and epileptic encephalopathy

**DOI:** 10.1002/epi4.12751

**Published:** 2023-05-05

**Authors:** Lin Wan, Dan Yu, Zhichao Li, Xinting Liu, Yan Liang, Huimin Yan, Gang Zhu, Bo Zhang, Guang Yang

**Affiliations:** ^1^ Department of Pediatrics, The Seventh Medical Center of PLA General Hospital Beijing China; ^2^ Department of Pediatrics, The First Medical Centre Chinese PLA General Hospital Beijing China; ^3^ Department of Pediatrics Medical School of Chinese People’s Liberation Army Beijing China; ^4^ Department of Pediatrics West China Second Hospital of Sichuan University Chengdu China; ^5^ Department of Neurology, ICCTR Biostatistics and Research Design Center, Boston Children's Hospital Harvard Medical School Massachusetts Boston USA; ^6^ Department of Pediatrics, The Second School of Clinical Medicine Southern Medical University Guangzhou China

**Keywords:** developmental and epileptic encephalopathies, malformations of cortical development, pathogenic, *RARS1* gene

## Abstract

**Objective:**

Biallelic variants of *RARS1*, a gene that encodes the cytoplasmic tRNA synthetase for arginine (ArgRS), are associated with central nervous system (CNS) manifestations, such as hypomyelinating leukodystrophy‐9 and developmental and epileptic encephalopathy (DEE). This study aimed to better understand the *RARS1* biallelic mutations and the associated phenotypes, particularly in patients with DEE.

**Methods:**

We identified two patients with *RARS1* biallelic mutations and functionally validated these mutations in vitro. Furthermore, we performed a review of the literature.

**Results:**

Two patients with hypomyelinating leukodystrophy were found to have *RARS1* biallelic variants (Patient 1: c.1535G>A (p.Arg512Gln) and c.1382G>A (p.Arg461His); Patient 2: homozygous variants c.5A>T (p.Asp2Val)). Patient 2 had a severe clinical manifestation of DEE. A review of the literature identified 27 patients from five studies. Among the 29 patients, intellectual disability, developmental delay, and hypomyelination were the common symptoms, while 13 of them exhibited DEE and malformations of cortical development. Of the 25 variants identified, c.5A>G (p.Asp2Gly) was identified in 10 patients. ArgRS protein expression and stability were substantially reduced in the two newly identified patients.

**Significance:**

Patients with *RARS1* biallelic mutations frequently exhibit DEE, a severe phenotype, along with hypomyelinating leukodystrophy. Besides its effects on the white matter, this mutation also influences cortical development. Moreover, the variants c.5A>T (p.Asp2Val), c.1382G>A (p.Arg461His), and c.1535G>A (p.Arg512Gln) are pathogenic and affect the expression of ArgRS by reducing the protein stability.

**Key points**

- *RARS1* biallelic variants could lead to DEE.
- All the patients with *RARS1*‐related DEE more likely to have MCD.
- The variants p.Asp2Val, p.Arg461His, and p.Arg512Gln of *RARS1* gene were pathogenic.

## 1 INTRODUCTION

Myelination, a process of compact myelin formation by oligodendrocytes, is crucial for the proper functioning of the central nervous system.^1^ Myelination plays a vital role in ensuring normal social cognitive, motor, sensory, and memory functions.^1^ Oligodendrogenesis and myelination are regulated by cell‐intrinsic mechanisms and extracellular signals, both during and after development.^1^ Impaired myelination may lead to detrimental activity patterns in neurological networks.^1^ The term hypomyelinating leukodystrophy (HLD) refers to a group of heritable neurodevelopmental disorders characterized by abnormal myelination in the central nervous system.^2^, ^3^ Hypomyelinating leukodystrophy has a broad clinical spectrum ranging from no to severe neurological symptoms in infants and adults.^4^, ^5^, ^6^, ^7^ With the incorporation of molecular genetic testing into clinical practice and due to technological advances and declining costs, several genes associated with HLD have been identified. Most of these genes, including the *RARS1* gene, do not encode structural myelin proteins.^3^, ^4^, ^5^

The *RARS1* gene encodes the cytoplasmic tRNA synthetase for arginine (ArgRS), a component of the multienzyme aminoacyl‐tRNA synthetase complex that is essential for protein synthesis.^8^ More than 20 patients with *RARS1*‐related HLD (known as HLD‐9 in OMIM, MIM#616140) have been reported. Intellectual disability (ID) or developmental delay (DD) was identified in the vast majority of these patients while nystagmus, epilepsy, and microcephaly were also common.^7^, ^8^, ^9^, ^10^, ^11^, ^12^

Since studies have shown that a broad spectrum of clinical symptoms are linked with *RARS1* variants, this study aimed to further investigate the relationships between genotype and/or phenotype in patients with HLD‐9. Also, we reviewed the literature and present two examples of patients with HLD9 who had biallelic *RARS1* mutations. Furthermore, *RARS1* variants from the two patients were functionally validated to verify their pathogenicity.

## 2 METHODS

### 2.1 Patients and literature review

Two unrelated children with hypomyelinating leukodystrophy carrying *RARS1* biallelic variants (identified by the trio‐WES) were enrolled, and their clinical and radiological data were retrospectively collected. Relevant studies were identified by two independent reviewers (LW and GY) by searching the electronic databases, including MEDLINE, PubMed, Embase, Google, and the China National Knowledge Infrastructure. The keyword search terms used were as follows: [RARS1 OR RARS OR arginine‐tRNA synthetase] AND [Hypomyelinating leukodystrophy OR Hypomyelinating leukodystrophy‐9 OR hypomyelination OR leukodystrophy] up to July 2022. We restricted the search to human studies published in English or Chinese. SPSS 26.0 statistical software was used for analysis. We used the chi‐squared and Fisher's exact tests to analyze the variables.

### 2.2 Functional validation

**FIGURE 1:**
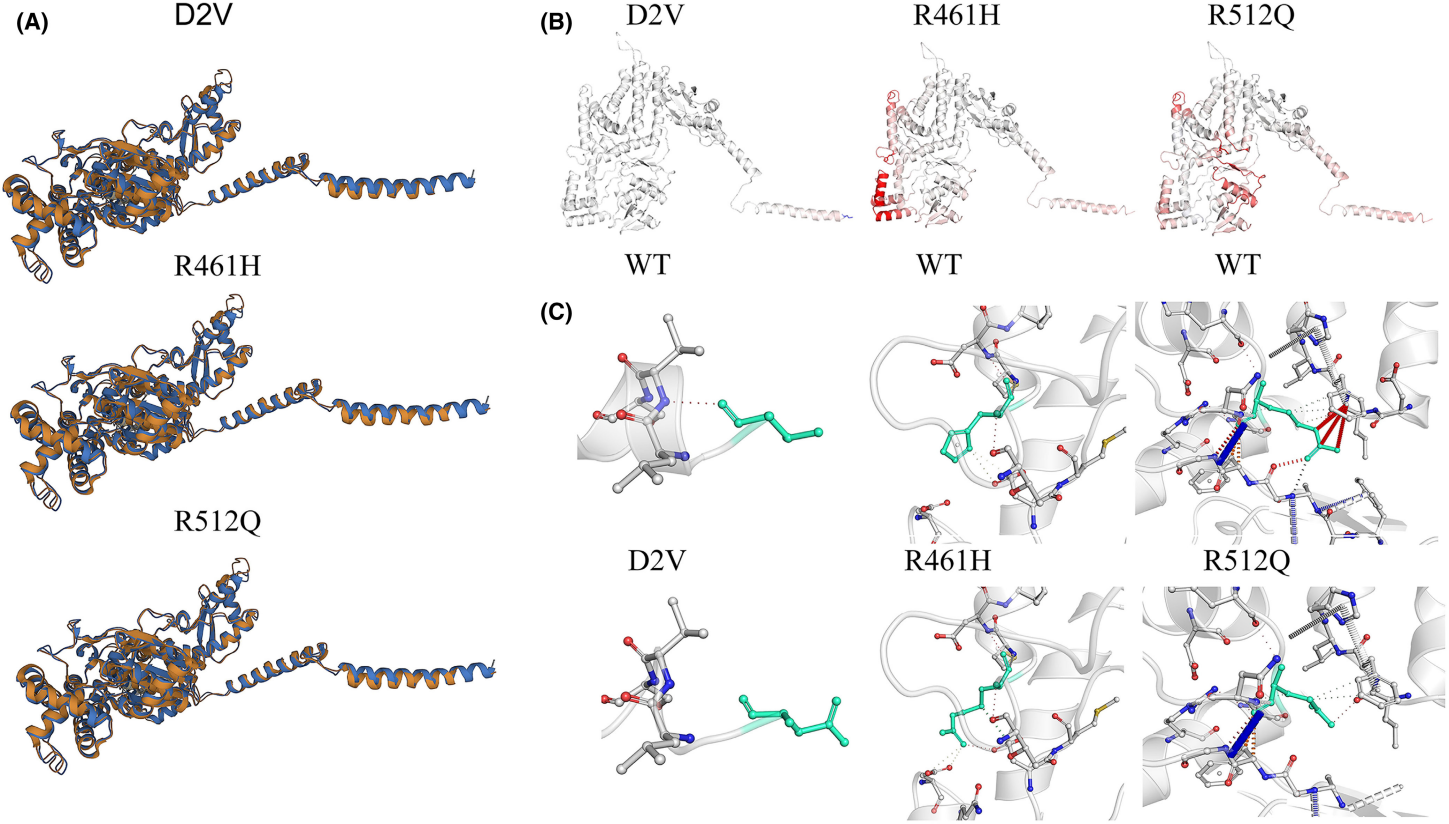
(A) jFATCAT‐rigid algorithms were used to perform pairwise structural alignments. The aligned regions of the two structures are shown in orange (wild‐type) and blue (Mutants). The root‐mean‐square deviation and template modeling score were well‐aligned between wild‐type and mutants. (B) Vibrational entropy energy between wild‐type and mutants showed significantly increased vibrational entropy energy in the mutants. Amino acids are colored according to the vibrational entropy change upon the variant. Blue represents a rigidification of the structure and red a gain in flexibility. (C) Prediction of interactomic interactions. The results showed the significant influence of interactomic interactions. Wild‐type and mutant residues are colored in light‐green and are also represented as sticks alongside the surrounding residues involved in any type of interaction. Red, Hydrogen bonds; Orange, Weak hydrogen bonds; Blue, Halogen bonds; Yellow, Ionic interactions.

The *RARS1* variants of the two patients were analyzed using some mutation‐prediction tools, including SIFT, PolyPhen‐2, MutationTaster, PROVEAN, and CADD to evaluate their pathogenicity. Results identified these variants as variants of unknown significance (VUS) according to the American College of Medical Genetics and Genomics (ACMG) guidelines. We obtained the protein structure template of RARS1 from the UniProt database via the AlphaFold website (www.uniprot.org) as wild‐type and mapped the corresponding mutations to generate mutated PDB files. We compared the mutated forms with the wild‐type using an online comparison method (fatcat.godziklab.org)^13^ and found that all mutant proteins exhibited a high similarity with the wild‐type (>99%; Table 1 and Figure 1A). Next, we used two online prediction software programs to analyze the effect of these mutations on protein stability. First, using the wild‐type PDB file as a template, we input the corresponding amino acid changes for each mutation in DynaMut (biosig.lab.uq.edu.au/dynamut)^14^ and observed an increase in entropy, leading to an increase in molecule flexibility. Additionally, we used DynaMut2 (biosig.lab.uq.edu.au/dynamut2)^15^ to input the corresponding amino acid changes for each mutation and found abnormal interactomics, causing reduced stability of the mutated protein (Table 1; Figure 1B,C). Our in silico results suggested that these mutations may affect the ArgRS protein function and structure, ultimately impacting its biological activity. We speculated that the patients' usual clinical symptoms might depend on the presence of specific variants and performed further research to confirm the effects of these mutations on ArgRS protein functions.

**TABLE 1:** Prediction of variants and the corresponding mutate protein.

Variant	SIFT	Polyphen2	MutationTaster	PROVEAN	CADD_raw	ACMG	RMSD	TM‐score	ΔΔG prediction outcome	Entropy energy
c.5A>T	Deleterious	Benign	Disease causing	Benign	1.912	VUS (PM2 + PM5 + PP3)	1.05	0.96	−0.600 kcal/mol (Destabilizing)	0.301 kcal/mol/K (Increase of molecule exibility)
c.1382G>A	Deleterious	Probably damaging	Disease causing	Deleterious	6.907	VUS (PM2 + PP3)	1	0.96	−0.483 kcal/mol (Destabilizing)	0.477 kcal/mol/K (Increase of molecule exibility)
c.1535G>A	Deleterious	Probably damaging	Disease causing	Deleterious	7.087	VUS (PM2 + PM5 + PP3)	1.06	0.96	−0.765 kcal/mol (Destabilizing)	0.656 kcal/mol/K (Increase of molecule exibility)

### 2.3 Vector construction

Using the cDNA sequence of *RARS1* (RefSeq: NM_004233.2), PCR amplification was performed using the following primer sets: phage‐RARS1‐SalI‐F, TGACGTCGACAATGGACGTACTGGTGTCTGA; phage‐RARS1‐NotI‐R, CGACGCGGCCGCTCATCCTTTGGACAGGTTTTA; RARS1‐variant1 c.5A>T‐F, TGACGTCGACAATGGTCGTACTGGTGTCTGA; RARS1‐mut1 c.1382.G>A‐F, TGACGTCGACAATGGACGTACTGGTGTCTGA; RARS1‐mut2 c.1382.G>A‐R, AGAAGATCCATGAGGTGCACTGTTTCACCCG; RARS1‐mut2 c.1535G>A‐F, ACCTTTCCCATAACCAGTTGAATGACTACAT; RARS1‐mut3 c.1535G>A‐ACCTTTCCCATAACCAGTTGAATGACTA; RARS1‐mut3 c.1535G>A‐R, ATGTAGTCATTCAACTGGTTATGGGAAAGGT. The PCR products were subjected to SalI and NotI digestion, cloned into the phage vector, and validated by sequencing. The vectors were then transfected into 293T cells using Lipo2000 (Thermo Fisher Scientific, Waltham, MA, USA).

### 2.4 *RARS1* and ArgRS expression

Cells were collected 48‐h posttransfection. The TRIzol method was used to extract total RNA. After DNA digestion, cDNA synthesis was performed by reverse transcription, and *RARS1* expression was validated by qPCR. Total protein was extracted from cells using the RIPA lysis buffer, and protein concentration was measured using the BCA Protein Quantification Kit (Yeasen Biotechnology, SH, China). Proteins were denatured, and ArgRS expression was confirmed by Western blot using the HA‐Tag monoclonal antibody (Dia‐an, WH, China).

### 2.5 ArgRS protein stability assay

Forty‐eight‐hour posttransfection, cells were treated with 500 μM cycloheximide and collected at 0, 4, 8, and 24 h after the treatment to confirm ArgRS protein expression by Western blot using the HA‐Tag monoclonal antibody (Dia‐an, WH, China).

## 3 RESULTS

### 3.1 Patients

**FIGURE 2:**
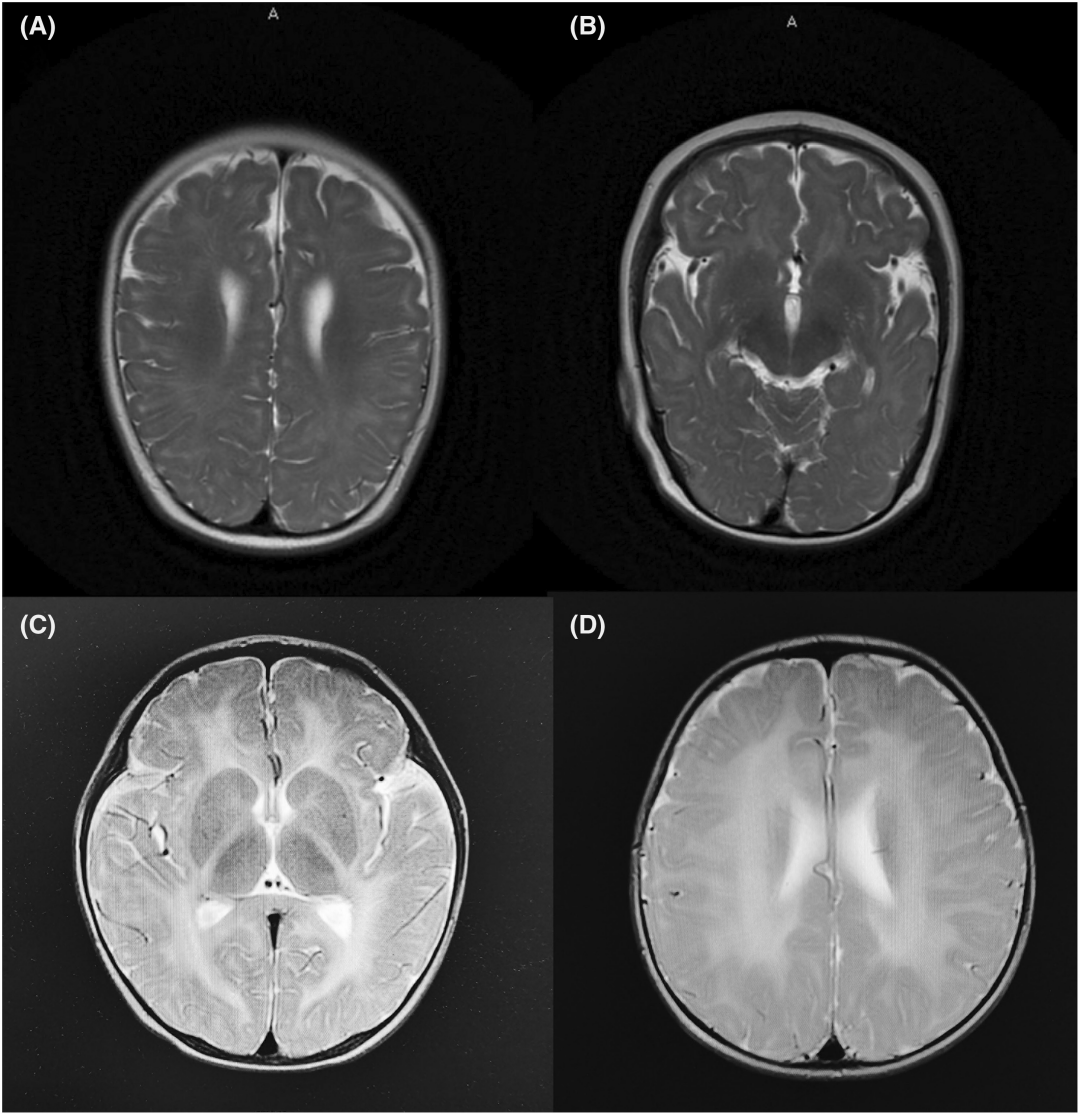
Brain MRI findings in patients with RARS1 biallelic variants. (A,B) Patient 1's axial T2‐weighted imaging showed an abnormal T2 hyperintense signal of the cerebral white matter and simplified gyral pattern. (C,D) Patient 2's axial T2‐weighted imaging showed an abnormal T2 hyperintense signal of cerebral white matter.

Patient 1, a 12‐month‐old girl, is the first child of unrelated, healthy parents with no family history of neurological disorders. She was born at term via spontaneous delivery with normal height and weight; congenital aural atresia was diagnosed during the postnatal period. Seizure onset as epileptic spasms occurred at 5 months of age. Hypsarrhythmia was observed on the electroencephalogram, and brain magnetic resonance imaging (MRI) showed hypomyelinating and a simplified gyral pattern, leading to the diagnosis of infantile spasms. Physical examination identified nystagmus and microcephaly, although ataxia and dystonia were absent. Adrenocorticotropic hormone and multiple antiseizure medications, including vigabatrin, were initiated, but these medications failed to control the problems. Brain MRI at 12 months showed hypomyelinating leukodystrophy and a simplified gyral pattern (Figure 2A,B).

The patient had severe developmental delay and exhibited partial head control at 3 months of age and started rolling over at 4 months of age; however, during her 12‐month follow‐up, she was still unable to sit. Her language development cannot be assessed effectively during this period.

Trio‐WES was performed and compound heterozygous variants c.1382G>A (p.Arg461His) and c.1535G>A (p.Arg512Gln) in *RARS1* (Figure S1A,B) were identified.

Patient 2, a 16‐month‐old girl, is the first child of unrelated, healthy parents with no family history of neurological disorders. She was born at term via spontaneous delivery with normal height and weight and exhibited no abnormalities during the postnatal period. Nystagmus and ataxia were diagnosed at 11 months of age. Brain MRI showed hypomyelinating leukodystrophy at 16 months of age (Figure 2C,D).

She had severe developmental delay, exhibited partial head control at 3 months of age, started rolling over at 4 months of age, and was able to sit unassisted at 12 months of age, although she was unable to walk at the 16‐month follow‐up. In addition, her language development was delayed and speech was absent.

Trio‐WES was performed and the homozygous variant c.5A>T (p.Asp2Val) in *RARS1* (Figure S1C) was identified.

### 3.2 Literature review and genotype/phenotype correlation

We reviewed the literature and identified 27 patients from five studies. Among the 29 patients carrying the *RARS1* biallelic variants (including the two newly reported in this study), 13 were female patients and 15 were male patients (one gender was not reported). Twenty‐five different variants were identified and variant c.5A>G (p.Asp2Gly) was present in 10 patients. The newly identified variants c.5A>T (p.Asp2Val) and c.1382G>A (p.Arg461His) were not reported in previous studies. Among the 25 variants, missense was the major type with a frequency of 13, followed by start codon defects (4), splicing (4), and frameshift (3).

Among the 29 patients, 21 exhibited nystagmus, 27 had intellectual disability or developmental delay, 22 had language delay (17 with absent speech), 25 exhibited motor delay, 17 had microcephaly, and 24 showed hypomyelination. Symptoms observed in less than half of the patients were ataxia (12 patients), dystonia (11 patients), hypotonia (8 patients), epilepsy (13 patients), and feeding difficulties (12 patients). Only eight patients were able to walk without any support (Table S1 and Figure S2A).

Of the 13 patients with epilepsy, all exhibited intellectual disability or developmental delay, 12 had seizure onset during infancy (1–13 months), 12 had hypomyelination (no results were reported for one patient), and 11 had microcephaly (no results were reported for one patient). Of the 12 patients who reported seizure outcomes, 11 developed drug‐resistant epilepsy (Table S1 and Figure S2B). Compared to patients without epilepsy, those with *RARS1*‐related epilepsy were more likely to have microcephaly, cerebral atrophy, and speech absence, but less likely to be able to walk independently (*P* < 0.05, Table 2).

**TABLE 2:** Phenotype comparation between epilepsy and nonepilepsy patients.

	Epilepsy group (*n* = 13) (percentage (case number/reported number)	Nonepilepsy group (*n* = 16) (percentage (case number/reported number)	*P* value
Nystagmus	69.2% (9/13)	75% (12/15)	0.67
Intellectual disability or developmental delay	100% (13/13)	87.5% (14/16)	0.488
Language delay	100% (11/11)	78.6% (11/14)	0.23
Speech absence	100% (11/11)	46.2% (6/13)	0.006
Motor delay	100 (13/13)	85.7% (12/14)	0.481
Walks with support	0 (0/13)	57.1% (8/14)	0.002
Microcephaly	100 (13/13)	40% (6/15)	0.001
Hypomyelination	100% (12/12)	85.7% (12/14)	0.483
Cerebral atrophy	91.7% (11/12)	42.9% (6/14)	0.014

Missense mutations, which result in the substitution of a single amino acid residue only, are less likely to produce aberrant transcripts, the majority of which may arise due to frameshift mutations, splicing defects, start codon mutations, etc. There were no significant differences in symptoms between patients with or without missense mutations only (*P* < 0.05, Table S2).

### 3.3 *RARS1* qPCR and Western blot for ArgRS

**FIGURE 3:**
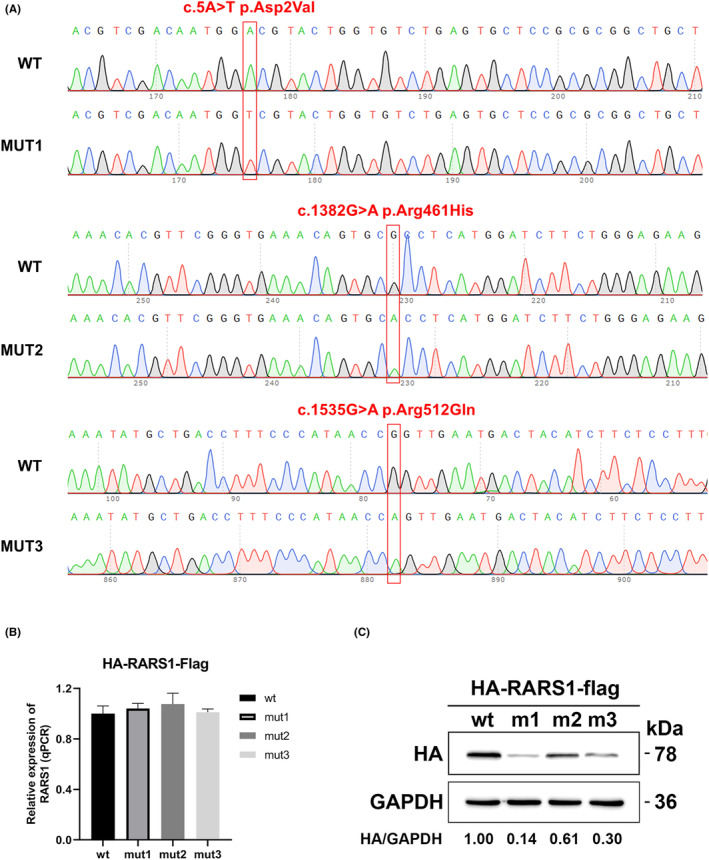
Expressions of RARS1 and ArgRS. (A) Sanger sequencing to confirm the vector was successfully constructed. (B) RARS1 expression confirmed by qPCR. (C) ArgRS expression confirmed by Western Blot. mut1/m1, c.5A>T; mut 2/m2, c.1382.G>A; mut3/m3, c.1535G>A.

We successfully developed the vector for *RARS1* overexpression (Figure 3A). There were no significant differences in *RARS1* mRNA expression between cells overexpressing wild‐type and mutant *RARS1* (Figure 3B). However, ArgRS protein expression was significantly lower in cells overexpressing *RARS1* variants (mut1 = 0.14, mut2 = 0.61, and mut3 = 0.30) compared to those expressing wild‐type *RARS1* (Figure 3C).

### 3.4 Protein stability assay

**FIGURE 4:**
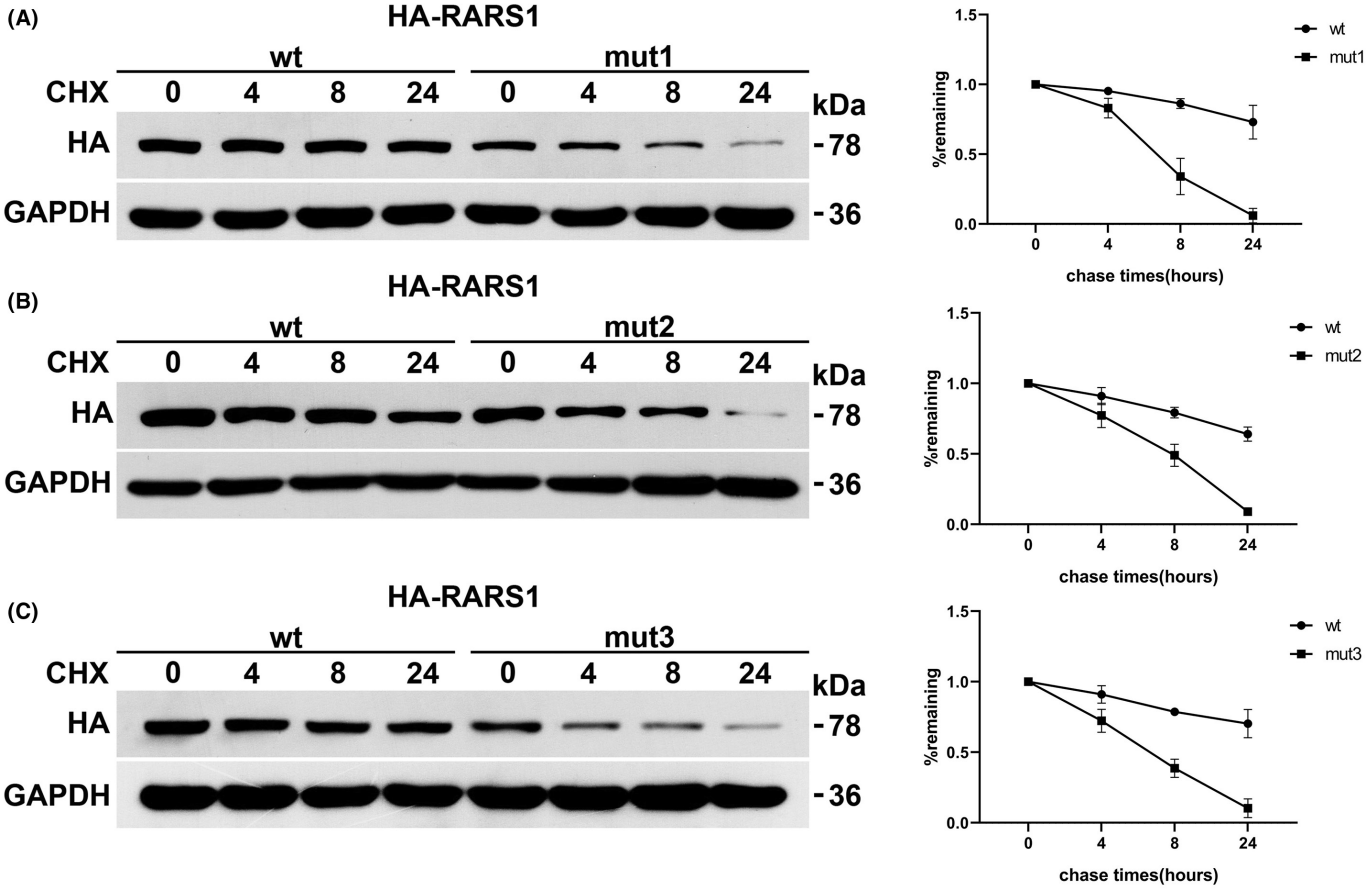
Protein stability assays of ArgRS. (A) MUT1, c.5A>T (B) MUT 2, c.1382.G>A. (C) MTU3, c.1535G>A.

At 0 h, ArgRS protein expression was significantly lower in cells overexpressing *RARS1* variants compared to those expressing wild‐type *RARS1* (Figure 4). Furthermore, ArgRS expression decreased as the time increased following cycloheximide treatment, indicating that protein degradation occurred. All the mutated ArgRS proteins degraded at a significantly higher rate compared with the wild‐type protein (Figure 4), indicating that these mutations decrease ArgRS stability.

## 4 DISCUSSION

Consistent with previous studies,^7^, ^8^ we found that *RARS1* biallelic variants can cause a range of neurological symptoms. Furthermore, intellectual disability/developmental delay and hypomyelination are the most prevalent phenotypes in the carriers of these variants. In the study by Mendes et al.,^7^ the clinical presentation was classified as severe, intermediate, or mild, with severe indicating that the patient developed symptoms, usually refractory epilepsy, during the first 3 months of life. In our study, Patient 1 presented the severe phenotype, with the onset of infantile spasms during infancy. After first‐line treatment with adrenocorticotropic hormone and vigabatrin, the spasms were not controlled, and the patient developed drug‐resistant epilepsy. Furthermore, a severe developmental delay was observed. This patient carried the c.1535G > A (p.Arg512Gln) variant, which is located close to the active center, and it has been reported in several previous studies that all patients with this variant exhibit the severe phenotype.^7^ Herein, we found that patients with epilepsy are more likely to exhibit severe phenotypes, such as speech absence and inability to walk. In these cases, the prognosis might be judged early based on the presence or absence of epilepsy.

Developmental and epileptic encephalopathies (DEE) are a heterogeneous group of disorders with both nongenetic and genetic etiologies and are characterized by early‐onset and often severe epileptic seizures and EEG abnormalities on a background of developmental impairment that tends to worsen as a consequence of epilepsy.^16^ In patients with the epilepsy phenotype, almost all seizures have been reported early during infancy (except in one case at 13 months of age). These patients with epilepsy had severe developmental delay and microcephaly and could be categorized as patients with DEE. Among these patients, other than the variants that led to protein truncation, such as those carrying splicing and frameshift defects, two missense variants [c.1535G>A (p.Arg512Gln) and c.1316C>A (p.Ala439Asp)] and one start codon defect variant (c.2T>C, p.Met1?) were present in multiple patients. This finding suggests a potential relationship between these variants and severe phenotypes.

We found that patients with epilepsy more often had microcephaly and cerebral atrophy. Microcephaly can be primary when it is congenital and presents at the time of birth or in utero as a small head with a simple gyral pattern,^17^ which is associated with a reduction in the number of gyri with shallow sulci (<50% of normal depth) but normal cortical thickness.^18^, ^19^ Microcephaly with a simplified gyral pattern is associated with impaired neurogenesis, resulting in a reduction in the number of neurons.^20^ Moreover, microcephaly with a simplified gyral pattern is a type of malformation of cortical development (MCD) and is often associated with neurological symptoms, such as epilepsy, cognitive impairment, and psychomotor retardation.^21^ In the study by Mendes et al.,^7^ patients who had microcephaly with a simplified gyral pattern exhibited severe DEE. Similarly, Patient 1 in our study had microcephaly with a simplified gyral pattern and exhibited DEE. Based on these results, we speculate that abnormalities in brain development caused by the *RARS1* variant occur not only in the white matter but also in the cortex and are presented as MCD. Therefore, patients with DEE are more likely to have MCD.

Using Western blot analysis, Mendes et al. confirmed that ArgRS protein is expressed in fibroblasts, and its expression was decreased in all four patients.^7^ The study also confirmed that the ArgRS enzyme activity was significantly lower in three patients compared with controls.^7^ Li et al. further verified the variants through in vitro experiments.^22^ The authors found that the *RARS1* gene carrying c.5A>G (p.Asp2Gly) and c.1535G>A (p.Arg512Gln) mutations produced ArgRS protein similar to the control; however, the decay rate of the ArgRS protein produced by the c.1535G>A variant was significantly increased. These results indicate that Arg512 is crucial for ArgRS enzyme activity and stability. In contrast, the c.1367C>T variant was associated with the production of ArgRS protein with normal stability and Arg activation ability; however, the enzyme exhibited a decreased aminoacylation activity.^22^ In our study, all three mutations resulted in a decrease in the intracellular ArgRS levels, which might be due to the decreased stability and consequently, increased decay rates of mutated ArgRS proteins. In the absence of ArgRS, the physiological processes cannot be effectively completed, thereby resulting in disease development. Notably, although the novel variant (c.5A>T) reported in this study and a previously reported variant (c.5A>G) both resulted in the substitution of the second amino acid Asp, the previously reported variant did not affect protein stability. These discrepancies in results suggest that the substituted amino acids might be different in the newly reported and previously reported variants.

In conclusion, we report two patients with *RARS1*‐related disorders and two novel *RARS1* variants. We confirmed that these mutations can reduce the stability of ArgRS protein and cause disease, including DEE. Furthermore, brain MRI examination in these patients with *RARS1*‐related DEE may indicate both hypomyelinating leukodystrophy and MCD. When a child experiences seizures during infancy and HLD is suspected based on MRI, it is necessary to consider testing for the *RARS1* gene as the patient may develop DEE in the future. Due to our limited knowledge of mutation sites, even a single variation classified as VUS by the ACMG guidelines requires additional functional validation to determine its potential pathogenicity.

## AUTHOR CONTRIBUTIONS

Lin Wan, Dan Yu, and Zhichao Li wrote the first draft of the manuscript and contributed equally to this work (cofirst authors). Dan Yu, Gang Zhu, and Guang Yang performed the acquisition of data. Yan Liang, Huimin Yan, and Bo Zhang performed the data analyses. Lin Wan and Yang Guang contributed to the conception and design of the study. All authors helped to revise the manuscript regarding crucial intellectual content. All authors approved the final version for publication.

## FUNDING INFORMATION

This research was funded by the General project of Beijing Natural Science Foundation (Reference: 7222187), the BINC foundation of Nutrition and Care of Maternal & Child (Reference: 2021BINCMCF030), the Epilepsy research foundation of Chinese Association Against Epilepsy (Reference: CU‐B‐2021‐11), and the Special scientific research project of military family planning (Reference: 22JSZ20).

## CONFLICT OF INTEREST STATEMENT

No financial or nonfinancial benefits have been received or will be received from any party related directly or indirectly to the subject of this article. We confirm that we have read the Journal's position on issues involved in ethical publication and affirm that this report is consistent with those guidelines.

## ETHICS APPROVAL AND CONSENT FOR PUBLICATION

Informed consent for clinical study and molecular genetic analysis was obtained from their parents. Ethics approval for the study was granted by the Ethics Committee of the First Medical Center of the PLA General Hospital.

## Supporting information


Data S1.

## Data Availability

The original sequencing data used to support the findings of this study are restricted by the Ethics Committee of the First Medical Center of the PLA General Hospital in order to protect patient privacy. Data are available from the corresponding author Guang Yang (yangg301@126.com) for researchers who meet the criteria for access to confidential data.
