# Supplementary material for: RARS1‐related developmental and epileptic encephalopathy
Source: Epilepsia Open. 2023 May 5;8(3):867–76. doi: 10.1002/epi4.12751 (PMC10472388; doi:10.1002/epi4.12751)

| Table S1. Genotype and phenotype of 29 patients with RARS1 biallelic variants. | | | | | | | | | | | | | | | | | | | | | | | | | |
| --- | --- | --- | --- | --- | --- | --- | --- | --- | --- | --- | --- | --- | --- | --- | --- | --- | --- | --- | --- | --- | --- | --- | --- | --- | --- |
| Patient | Age(years) | Gender | Genotype | | | | | | Phenotype | | | | | | | | | | | | | | | Main MRI findings | |
|  |  |  | Variant 1 | Type | Location | Variant 2 | Type | Location | Nystagmus | Ataxia | Dystonia | Microcephaly | Hypotonia | Intellectual disability/Development delay | Language delay | Speech absence | Motor delay | Sitting without support | Walking | Highest motor milestone | Epilepsy | Epilepsy outcome | Feeding difficulties | Hypomyelination | Cerebral atrophy |
| Ji et al.(patien118) | N/A | F | c.5A>G, p.Asp2Gly | Missense | Exon 1 | c.1625+2T>G p.(?) | Splicing | intron between exon 13 and 14 | Y | N/A | N/A | N/A | Y | Y | N/A | N/A | N/A | N/A | N/A | N/A | N | N/A | N | Y | Y |
| McSherryet al.(patient s224) | 8 months | M | c.1588A>G, p.Thr530Ala | Missense | Exon 13 | c.1588A>G,p.(Thr530Ala) | Missense | Exon 13 | N/A | N/A | N | Y | N | Y | N/A | N/A | N/A | N/A | N/A | N/A | N/A | N/A | N/A | N/A | N/A |
| Mendes et al.(Patient 1) | 11 | F | c.5A>G, p.Asp2Gly | Missense | Exon 1 | c.45+1G>T p.(?) | Splicing | Intron between exon 1 and 2 | Y | Y(36 months) | Y | N | N | Y | N | N | Y | N/A | Walks with support(2 years) | Walks with support | N | N/A | N | Y | N |
| Mendes et al.(Patient 2) | 8 | F | c.5A>G, p.Asp2Gly | Missense | Exon 1 | c.45+1G>T p.(?) | Splicing | Intron between exon 1 and 2 | Y | Y(20 months) | Y | N | N | Y | Y | N | Y | Y | N | Crawls | N | N/A | N | Y | N |
| Mendes et al.(Patient 3) | 26 | M | c.5A>G, p.Asp2Gly | Missense | Exon 1 | c.96_97del,p.(Cys32Trpfs*39) | Frameshift | Exon 2 | Y | Y(24-36 months) | Y | N | N | Y | Y | N | Y | Y | Walks with support(4 years) | Walks with support | N | N/A | N | Y | N |
| Mendes et al.(Patient 4) | 8 | F | c.1A>G, p.Met1? | Start codon defect | Exon 1 | c.1535G>A,p.(Arg512Gln) | Missense | Exon 13 | Y | N | Y | Y | Y | Y | Y | Y | Y | N | N | Rolls over | N | N/A | Y | Y | Y |
| Mendes et al.(Patient 5) | 1 | M | c.1316C>A, p.Ala439Asp | Missense | Exon 12 | c.1316C>A,p.(Ala439Asp) | Missense | Exon 12 | Y | N | N | Y | Y | Y | Y | Y | Y | N | N | Partial head contro | Y(Infancy) | Refractory | Y | Y | Y |
| Mendes et al.(Patient 6) | 1 | M | c.1316C>A, p.Ala439Asp | Missense | Exon 12 | c.1316C>A,p.(Ala439Asp) | Missense | Exon 12 | N | N | N | Y | Y | Y | Y | Y | Y | N | N | Partial head control | Y(4months) | Refractory | Y | N/A | N/A |
| Mendes et al.(Patient 7) | 3 | M | c.173T>C, p.Leu58Pro | Missense | Exon 2 | c.1790T>C p.(Leu597Pro) | Missense | Exon 14 | Y | Y(4 months) | N | Y | N | Y | Y | Y | Y | N | N | N | Y(Neonate) | Refractory | Y | Y | Y |
| Mendes et al.(Patient 8) | 9 | M | c.5A>G, p.Asp2Gly | Missense | Exon 1 | c.1874-9_1874-5del p.(?) | Splicing | Intron between exon 14 and 15 | Y | Y(4 years) | N | N | N | Y | Y | Y(3-4 words phrases) | Y | Y | N | Sits without support | N | N/A | N | Y | Y |
| Mendes et al.(Patient 9) | 11 | M | c.5A>G, p.Asp2Gly | Missense | Exon 1 | c.1874-9_1874-5del p.(?) | Splicing | Intron between exon 14 and 15 | Y | Y(4 years) | N | N | N | Y | Y | Y(3-4 words phrases) | Y | Y | N | Sits without support | N | N/A | N | Y | Y |
| Mendes et al.(Patient 10) | 55 | N/A | c.5A>G, p.Asp2Gly | Missense | Exon 1 | c.5A>G p.(Asp2Gly) | Missense | Exon 1 | Y | N | N | N | N | Y | N | N | N | Y | Y | Walks without support | N | N/A | N | Y | Y |
| Mendes et al.(Patient 11) | 21 months | F | c.67_70r23Leufs*6 | Frameshift | Exon 2 | c.67_70del,p.(Thr23Leufs*6) | Frameshift | Exon 2 | Y | N | Y | Y | N | Y | Y | Y | Y | N | N | Partial head contro | Y(Infancy) | Refractory | Y | Y | Y |
| Mendes et al.(Patient 12) | 21 months | F | c.67_70del,p.Thr23Leufs*6 | Frameshift | Exon 2 | c.67_70del,p.(Thr23Leufs*6) | Frameshift | Exon 2 | Y | N | Y | Y | N | Y | Y | Y | Y | N | N | Partial head contro | Y(Infancy) | Refractory | Y | Y | Y |
| Mendes et al.(Patient 13) | 14 | F | c.668G>A,p.Arg223His | Missense | Exon 6 | c.1568T>A p.(Met523Lys) | Missense | Exon 13 | N | N | N | N | N | Y | Y | Y(3 words) | Y | Y | Walks without support(3.5 years) | Walks without support | N | N/A | Y | N | N |
| Mendes et al.(Patient 14) | 6 | F | c.5A>G, p.Asp2Gly | Missense | Exon 1 | c.173T>C p.(Leu58Pro) | Missense | Exon 2 | N | Y(3 years) | N | N | Y | N | Y | N | Y | Y | Walks without support(2 years) | Walks without support | N | N/A | N | Y | N |
| Mendes et al.(Patient 15) | 2 | M | c.2T>A, p.Met1? | Start codon defect | Exon 1 | c.448_456del,p.(Cys150_Glu152del) | Inframe | Exon 2 | N | N | N | Y | N | Y | Y | Y | Y | N | N | Partial head contro | Y(4months) | Refractory | Y | Y | Y |
| Mendes et al.(Patient 16) | 7 | M | c.2T>C, p.Met1? | Start codon defect | Exon 1 | c.1535G>A,p.(Arg512Gln) | Missense | Exon 13 | Y | N | N | Y | N | Y | Y | Y | Y | N | N | Rolls over, partial head control | Y(12months) | Controlled | Y | Y | Y |
| Mendes et al.(Patient 17) | 3.5 | M | c.1452+1G>A, p.? | Splicing | Intron between exon 13 and 13 | c.1534C>T p.(Arg512Trp) | Missense | Exon 13 | N | N | Y | Y | N | Y | Y | Y | Y | N | N | N | Y(2months) | Refractory | Y | Y | Y |
| Mendes et al.(Patient 18) | 12 months | M | c.1452+1G>A, p.? | Splicing | Intron between exon 12 and 13 | c.1534C>T p.(Arg512Trp) | Missense | Exon 13 | Y | N | N | Y | N | Y | Y | Y | Y | N | N | N | Y(Neonate) | Refractory | Y | Y | Y |
| Mendes et al.(Patient 19) | 2 | F | c.3G>T, p.Met1? | Start codon defect | Exon 1 | c.96_97del,p.(Cys32Trpfs*39) | Frameshift | Exon 2 | N | N | N | Y | N | Y | Y | Y | Y | N | N | N | Y(Neonate) | Refractory | Y | Y | Y |
| Mendes et al.(Patient 20) | 10 | F | c.475C>T,p.Pro159Ser | Missense | Exon 5 | c.1367C>T p.(Ser456Leu) | Missense | Exon 12 | N | N | N | N | N | N | Y | Y (Auditory processing issues) | N | Y | Walks without support(17 months) | Walks without support | N | N/A | N | N | N |
| Nafisinia et al. (patient 1) | 17 | M | c.5A>G, p.Asp2Gly | Missense | Exon 1 | c.5A>G p.(Asp2Gly) | Missense | Exon 1 | Y | Y(6 years) | N | N | N | Y | Y | N | Y | Y | Walks without support(2.5 years) | Walks without support | N | N/A | N | Y | Y |
| Nafisinia et al. (patient 2) | 15 | F | c.5A>G, p.Asp2Gly | Missense | Exon 1 | c.5A>G p.(Asp2Gly) | Missense | Exon 1 | Y | Y(4 years) | Y | N | N | Y | N | N | Y | Y | Walks without support(2 years) | Walks without support | N | N/A | N | N/A | N/A |
| Nafisinia et al. (patient 3) | 18 | M | c.1367C>T, p.Ser456Leu | Missense | Exon 12 | c.1846_1847del,p.(Tyr616Leufs*6) | Frameshift | Exon 14 | Y | N | Y | Y | Y | Y | Y | Y | Y | Y | N | Sitting without support | Y(10months) | Refractory | N/A | Y | Y |
| Rezaei et al.(patient 1) | 10 | F | c.2T>C, p.Met1? | Start codon defect | Exon 1 | c.2T>C p.(Met1?) | Start codon defect | Exon 1 | Y | Y | N | Y | Y | Y | Y | N/A | Y | N | N | Head control | N | N/A | N/A | Y | N |
| Rezaei et al.(patient 2) | 2 | M | c.2T>C p.Met1? | Start codon defect | Exon 1 | c.2T>C p.(Met1?) | Start codon defect | Exon 1 | Y | Y | Y | Y | Y | Y | N/A | N/A | Y | N | N | Head control | Y(13 months) | Unkown | N/A | Y | Y |
| Current study Patient 1 | 14 months | F | c.1535G>A, p.Arg512Gln | Missense | Exon 13 | c.1382G>A (p.Arg461His) | Missense | Exon 12 | Y | N | N | Y | N | Y | N/A | N/A | Y | N | N | Partial head control,Rolls over | Y(5 months) | Refractory | N | Y | N |
| Current study Patient 2 | 16 months | M | c.5A>T, p.Asp2Val | Missense | Exon 1 | c.5A>T (p.Asp2Val) | Missense | Exon 1 | Y | Y (11 months) | Y | N | N | Y | Y | Y | Y | Y | N | Sits without support | N | N/A | N | Y | N |
| N/A, not report or not applied. | | | | | | | | | | | | | | | | | | | | | | | | | |

| Table S2. Phenotype comparation between patients with only missense or other mutations. | | | |
| --- | --- | --- | --- |
|  | Only missense（n=14）  ([Percentage](javascript:;) (case number/reported number) | With other type（n=15）  ([Percentage](javascript:;) (case number/reported number) | P value |
| Nystagmus | 69.2%（9/13） | 80%(12/15) | 0.67 |
| Ataxia | 53.8%（7/13） | 35.7%（5/14） | 0.449 |
| Dystonia | 21.4%（3/14） | 57.1%（8/14） | 0.12 |
| Intellectual disability or developmental delay | 85.7%（12/14） | 100%（15/15） | 0.224 |
| Language delay | 81.8%(9/11) | 92.9%(13/14) | 0.565 |
| Speech absence | 60%(6/10) | 78.6%(11/14) | 0.393 |
| Motor delay | 84.6%(11/13) | 100%(14/14) | 0.222 |
| Walks without support | 46.2(6/13) | 14.3%(2/14) | 0.103 |
| Microcephaly | 50%（7/14） | 64.3%（9/14） | 0.704 |
| Epilepsy | 35.7%(5/14) | 53.4%(8/15） | 0.462 |
| Hypomyelination | 81.8%(9/11) | 100%(15/15) | 0.169 |
| Cerebral atrophy | 45.5%(5/11) | 80%(12/15) | 0.103 |

Figure S1. Pedigree and sequencing result. (A) variant c.1535G>A. (B) variant c.1382.G>A. (C) variant c.5A>T.





Figure S2. Phenotype of 29 patients with RARS1 biallelic variants(A) and 13 patients with epilepsy(B).


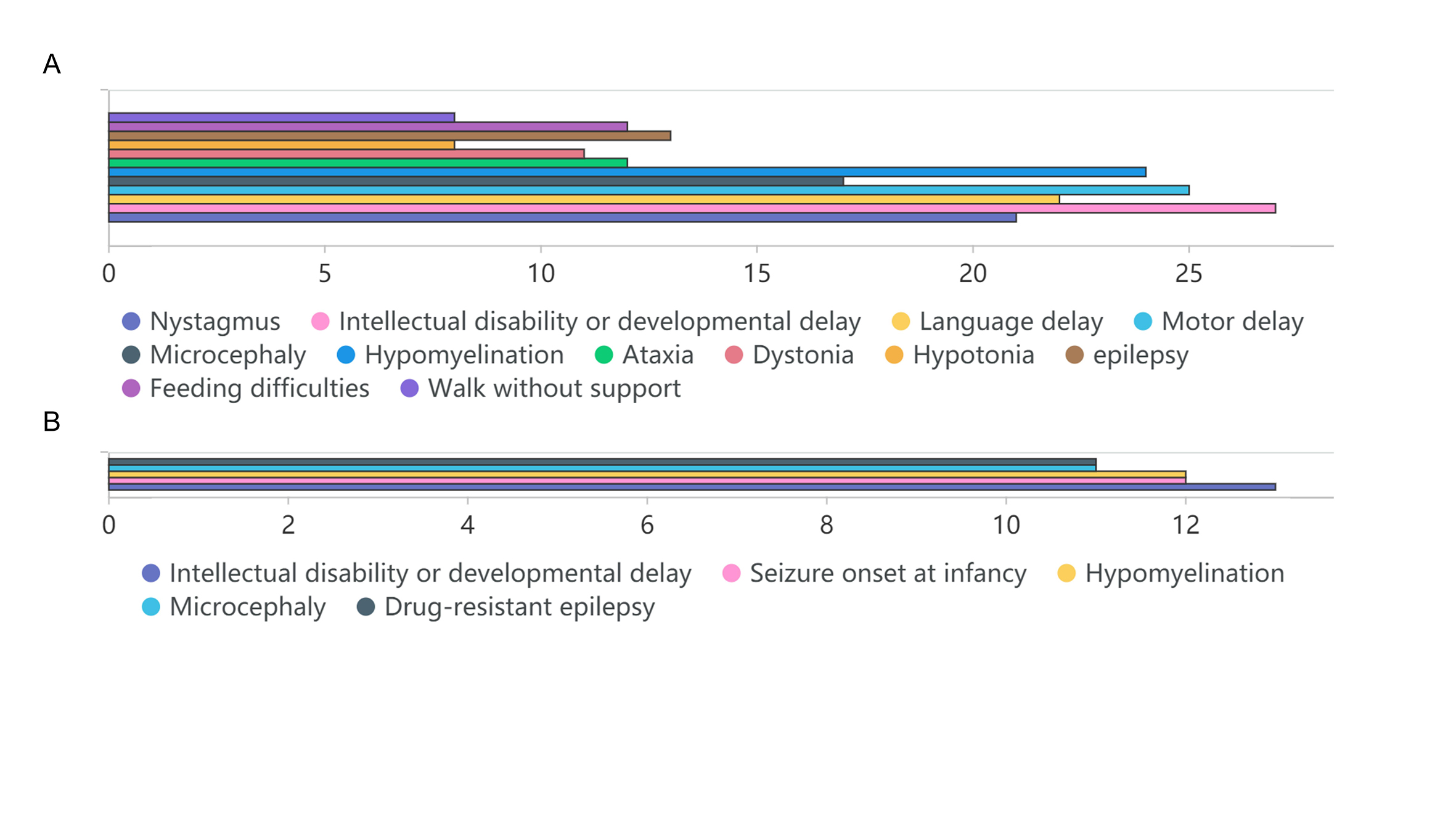

Supplement: Supplementary file 1 — Data S1. [file EPI4-8-867-s001.docx]
